# L'abcès fessier: une complication inhabituelle du cancer du rectum

**DOI:** 10.11604/pamj.2014.17.312.4399

**Published:** 2014-04-25

**Authors:** Souiki Tarik, Benjelloun El Bachir

**Affiliations:** 1 Service de Chirurgie Viscérale, Département de Chirurgie, Faculté de Médecine et de Pharmacie de Fès, Université Sidi Mohammed Ben Abdellah, CHU Hassan II, Fès, Maroc

**Keywords:** Cancer rectal, abcès gluteal, rectal cancer, gluteal abscess

## Image en medicine

La perforation sous-péritonéale du cancer du rectum est rare. Sa découverte est souvent tardive par une infection périnéale sévère, nécessitant un diagnostic rapide et une prise en charge médico-chirurgicale urgente. Nous rapportons l'observation d'un patient présentant une perforation sous péritonéale d'un cancer rectal révélée par un abcès de la région fessière. Il s'agit d'un patient âgé de 44 ans, chez qui un adénocarcinome du bas rectum non métastatique a été récemment découvert, et qui consulte pour une tuméfaction douloureuse de la fesse droite apparu depuis une semaine et évaluant dans un contexte de fièvre et d'altération de l’état général. L'examen clinque note une tuméfaction rénitente chaude et sensible du quadrant supero-externe de la fesse droite d'environ 10 cm de diamètre (A). Le toucher rectal met en évidence un processus tumoral bourgeonnant circonférentiel situé à 2 cm de la marge anale. Le bilan biologique montre un syndrome infectieux franc: hyperleucocytose à 15000 /mm et C-réactive protéine à 144mg/dl. Le scanner abdomino-pelvien objective un énorme processus tumoral rectal nécrosé et perforé (astérisque) en sous péritonéal et compliqué d'une collection abcédée de l'excavation pelvienne; cet abcès fuse vers la région fessière à travers l’échancrure ischiatique (flèche) (B, C). Par ailleurs, il n'existe pas d’épanchement intra-péritonéal. Notre patient a été mis sous antibiothérapie à large spectre. Un drainage chirurgical de l'abcès à travers une incision en regard de la tuméfaction fessière a été effectué en urgence, ramenant 300 de pus francs et de débris tumoraux. Une sigmoidostomie de dérivation du flux fécal à été associée par voie élective. Quant au traitement du cancer rectal, il ne sera envisagé que secondairement, après avoir complètement contrôlé l'infection.

**Figure 1:**
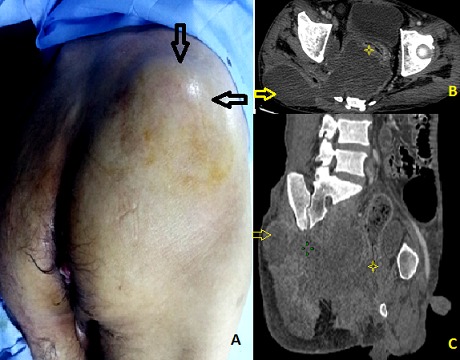
A) photo montrant un abcès de la région fessière droite (flèche); (B,C) Coupe scanographique (B) avec reconstruction (C) montrant une tumeur du bas rectum perforée (astérisque) à l'origine d'une collection abcédée de l'excavation pelvienne fusant vers la région fessière droite à travers l’échancrure ischiatique

